# Supplementary material for: Luminescent Papers with Asymmetric Complexes of Eu(III) and Tb(III) in Polymeric Matrices and Suggested Combinations for Color Tuning
Source: Molecules. 2023 Aug 21;28(16):6164. doi: 10.3390/molecules28166164 (PMC10459821; doi:10.3390/molecules28166164)
Supplement: Supplementary file 1 [file molecules-28-06164-s001.zip › molecules-2552685-supplementary.pdf]

Supplementary File

Luminescent Papers with Asymmetric Complexes of Eu(III) and Tb(III) in Polymeric Matrices, and Suggested Combinations for Color Tuning

Roberto J. Aguado [1], Beatriz O. Gomes [2], Luisa Durães [3], Artur J.M. Valente [2,*]

1 LEPAMAP-PRODIS Research Group, University of Girona, M. Aurèlia Capmany 61, 17003 Girona, Spain
2 University of Coimbra, CQC, Department of Chemistry, Rua Larga, 3004-535 Coimbra, Portugal
3 University of Coimbra, CIEPQPF, Department of Chemical Engineering, Rua Sílvio Lima, 3030-790 Coimbra, Portugal
* Correspondence: avalente@ci.uc.pt

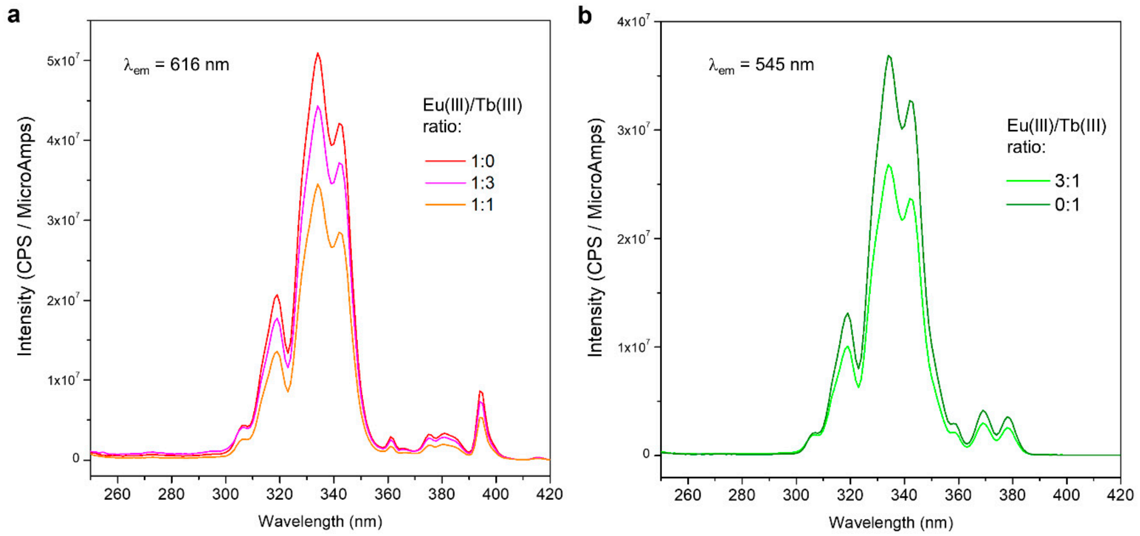

Figure S1: Excitation spectrum of Ln(III)/PSA/Phen, monitored either at 616 nm (a) or 545 nm (b).

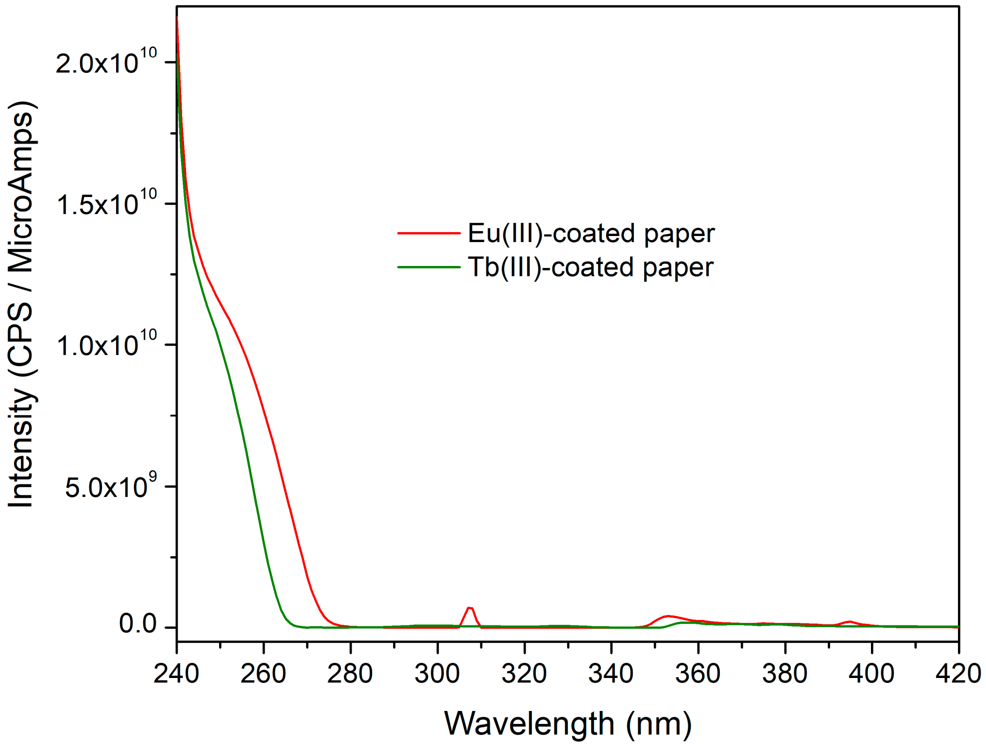

Figure S2: Excitation spectrum of Ln(III)-coated papers, monitored either at 616 nm (europium) or 545 nm (terbium)
